# Optogenetic Inhibition of Na_v_1.8 Expressing Corneal Afferents Reduces Persistent Dry Eye Pain

**DOI:** 10.1167/iovs.62.14.15

**Published:** 2021-11-17

**Authors:** Neal E. Mecum, Rachel Russell, Jun Lee, Cara Sullivan, Ian D. Meng

**Affiliations:** 1 Center for Excellence in the Neurosciences, University of New England, Biddeford, Maine, United States; 2 Molecular and Biomedical Sciences, University of Maine, Orono, Maine, United States; 3 Department of Complete Denture Prosthodontics, School of Dentistry, Nihon University, Tokyo, Japan; 4 Graduate Studies in Biomedical Sciences and Engineering, University of Maine, Orono, Maine, United States; 5 Department of Biomedical Sciences, College of Osteopathic Medicine, University of New England, Biddeford, Maine, United States

**Keywords:** dry eye, lacrimal gland, nociception, pain, cornea

## Abstract

**Purpose:**

The aim of the present study was to investigate the contribution of Nav1.8 expressing corneal afferent neurons to the presence of ongoing pain in lacrimal gland excision (LGE)-induced dry eye.

**Methods:**

The proton pump archaerhodopsin-3/eGFP (ArchT/eGFP) was conditionally expressed in corneal afferents using Nav1.8-cre mice. Dry eye was produced by unilateral LGE. Real time place preference was assessed using a three-chamber apparatus. A neutral, unlit center chamber was flanked by one illuminated with a control light and one illuminated with an ArchT activating light. For real-time preference, animals were placed in the neutral chamber and tracked over five 10-minute sessions, with the lights turned on during the second and fourth sessions. In other studies, movement was tracked over three 10-minute sessions (the lights turned on only during the second session), with animals tested once per day over the course of 4 days. A local anesthetic was used to examine the role of ongoing corneal afferent activity in producing place preference.

**Results:**

The corneal afferent nerves and trigeminal ganglion cell bodies showed a robust eGFP signal in Nav1.8-cre;ArchT/eGFP mice. After LGE, Nav1.8-cre;ArchT/eGFP mice demonstrated a preference for the ArchT activating light paired chamber. Preference was prevented with pre-application to the cornea of a local anesthetic. Nav1.8-cre;ArchT/eGFP mice with sham surgery and LGE wild-type control mice did not develop preference.

**Conclusions:**

Results indicate LGE-induced persistent, ongoing pain, driven by Nav1.8 expressing corneal afferents. Inhibition of these neurons represents a potential strategy for treating ongoing dry eye-induced pain.

Dry eye disease (DED) is a multifactorial disease of the ocular surface often, but not always, presenting with signs of inflammation and damage to the ocular surface.^1^^,^^2^ Although the most common clinical presentations of DED include feelings of ocular discomfort, irritation, and pain, both corneal hyperesthesia and hypoesthesia have been reported when assessing corneal sensitivity in patients with DED.^3^^–^^7^ These somewhat contradictory observations suggest that spontaneous ongoing corneal pain, rather than evoked pain, might be most relevant in assessing corneal pain in preclinical models.

In rodents, mechanical sensitivity of the cornea can be assessed by the presence of an evoked response, consisting of blinking, eye retraction, or head movement, to the application of a calibrated monofilament.^8^^–^^13^ Corneal sensitivity to noxious chemicals, such as capsaicin or hypertonic saline, can be assessed by quantifying eye wipe behaviors.^8^^,^^14^^–^^16^ Based on these evoked mechanical and chemical responses, dry eye models in the rat and mouse have reported an increase, decrease, or no change in corneal sensitivity.^8^^–^^16^ In contrast to evoked pain, spontaneous ongoing pain can be experienced in the absence of a specific applied stimulus.^17^ Signs of ongoing ocular pain produced by lacrimal gland excision (LGE)-induced dry eye have been shown in the rat through an increase in blink rate^8^^,^^18^^–^^21^ and in the mouse through a decrease in the palpebral opening.^9^^,^^15^ Both of these effects observed after LGE were reversed by the application of local anesthetics to the cornea, indicating that primary afferent nociceptors were driving these behaviors.^8^^,^^15^

Although increased blink rate or decreased palpebral opening have been used as signs of ongoing pain, as brainstem motor reflexes, they may not accurately reflect the sensory or affective components of the pain experience.^22^^,^^23^ The affective dimension of pain can be assessed by its ability to act as a teaching signal, producing a conditioned place aversion when presented within a specific context.^24^ Similarly, relief of pain in the presence of ongoing pain can be detected by its rewarding properties and ability to produce a conditioned place preference.^25^ Using an optogenetic approach that allows for temporal control of nociceptor activity, the selective activation of Nav1.8-positive primary afferent neurons using Nav1.8-cre;channelrhodopsin-2 (ChR2) transgenic mice produced a place aversion.^26^ Furthermore, optogenetic inhibition of peripheral terminals of calcitonin gene-related peptide-alpha (CGRP-alpha) positive primary afferent neurons using the conditional expression of the outward proton pump archaerhodopsin-3 produced a place preference in mice after spinal nerve ligation.^27^

Previously, we utilized a Nav1.8-cre;tdTomato reporter mouse to investigate corneal afferent nerve density changes following LGE.^15^ The voltage gated sodium channel Nav1.8 is expressed in most C-fibers and a portion of large diameter primary afferent neurons.^28^ Nav1.8-cre;tdTomato mice showed robust and extensive labeling of the corneal afferent subbasal nerve and intraepithelial nerve terminals.^15^ In the present study, the Nav1.8-cre mouse was used to conditionally express archaerhodopsin-T (ArchT), which inhibits peripheral nerve terminals when activated by a green/yellow light.^27^^,^^29^ Using this approach, the rewarding effects of corneal afferent inhibition was examined in dry eye.

## Methods

### Animals

Male and female C57BL/6J mice aged 8 to 10 weeks were obtained from Jackson Laboratory (Bar Harbor, ME, USA). A Nav1.8-cre mouse line,^30^ generously provided by Sulayman D. Dib-Hajj (Yale University, New Haven, CT) and rederived from mice with a C57BL/6J background, was used to selectively express ArchT in nociceptors. In a previous study, Nav1.8-cre;tdTomato mice were shown to exhibit robust labeling of corneal afferents, including stromal nerve bundles, subbasal nerve plexus, and intraepithelial nerve endings.^15^ Ai40(RCL-ArchT/eGFP)-D (ArchT) mice were obtained from Jackson Laboratory (stock # 021188) and ArchT/eGFP was conditionally expressed by crossing with Nav1.8-cre mice. Animals were housed in a controlled 12-hour light/dark cycle (07:00 lights on/19:00 lights off), temperature 20–22°C, 40–50% humidity, with free access to food and water, and treated according to the policies and recommendations of the National Institutes of Health guidelines for the handling and use of laboratory animals and in accordance with the ARVO Statement for the Use of Animals in Ophthalmic and Vision Research. All procedures were approved by the Institutional Animal Care and Use Committee at the University of New England.

### Surgery

Surgeries to excise the lacrimal glands were performed as previously described.^15^^,^^31^ Briefly, under isoflurane anesthesia, a unilateral LGE was performed, excising both the extraorbital and intraorbital glands. For sham surgeries, incisions were made to partially expose both the extra- and intraorbital glands. Mice were tested 2 weeks after surgery.

### Tissue Processing

Animals were perfused with 4% paraformaldehyde (PFA) and corneas and trigeminal ganglia were harvested. Trigeminal ganglia were post-fixed overnight in PFA and placed in 30% sucrose prior to cutting 12 µm sections on a cryostat; corneas were post-fixed in PFA for 1 hour prior and placed in 30% sucrose prior to staining. For immunohistochemistry, sections were briefly washed and then blocked with 5% normal donkey serum (NDS) in phosphate buffered saline (PBS)-Triton X-100 for 1 hour in a humidity chamber. Primary antibodies for CGRP (1:750, 1720–9007, BioRad) or PGP9.5 (1:1000, ab108986; Abcam) were applied to the slides or free-floating corneas overnight at 4C. Corneas were post-fixed in PFA for 1 hour prior to mounting. Trigeminal ganglia were post-fixed overnight in PFA and placed in 30% sucrose prior to cutting 12 µm sections on a cryostat. For immunohistochemistry, sections were briefly washed and then blocked with 5% NDS in PBS Triton X-100 for 1 hour in a humidity chamber. Primary antibodies for CGRP (1:750, 1720–9007, BioRad) were applied to the slides overnight at 4°C. After some brief washes with PBS Triton X-100, secondary antibody (1:200, Alexa Fluor 568 anti-rabbit) was applied for 1 hour. The isolectin IB4 was labeled by adding Alexa Fluor 647 conjugated IB4 (1:200, I32450; ThermoFisher) with the secondary antibody. Tissue was then washed with PBS, 3 × 5 minutes and coverslipped using Vectashield mounting medium with DAPI (Vector Labs, Burlingame, CA, USA).

### Imaging

Images of trigeminal ganglia sections were taken using a Leica DM 2500M microscope with a Leica DFC 365FX camera at 40 times magnification. Maximum intensity projections from widefield z-stacks were taken with the Keyence BZ-X710 (Itasca, IL, USA) using a Nikon fluorite corrected 40x/0.75 NA lens, 1.0 µm step size for corneal nerves. Pixel intensity quantification was performed as previously described.^15^

### Real Time Place Preference

A real-time active learning paradigm was performed using a three-chambered apparatus (outside chambers, 14 cm × 23 cm; inside chamber, 10 cm × 13 cm). The neutral center chamber was flanked by one illuminated with a control LED light source (wavelength = 360–400 nm, 35–45 lux) and one illuminated with ArchT activating LED light (560–610 nm, 35–45 lux). Animals were placed in a neutral box (one chamber gray walls, one chamber horizontal black/white stripe walls; odor-free with same texture floors) and movement was tracked over a 50-minute session (EthoVision XT 11.5.1020; Noldus Information Technology). The session was divided into five 10-minute periods. The LED lights were off during the first (baseline), third, and fifth periods. The control and ArchT activating LED lights were turned on during the second period. In the fourth period, the LED lights were again turned on, but the wavelengths were switched so that the chamber paired with the ArchT activating light during the second period was illuminated with the control light source.

### Conditioned Place Preference

Place preference learning over 4 consecutive days was assessed using the same 3-chambered real time place preference apparatus. Animals were placed in the neutral chamber and movement was tracked over a 30-minute session. The time was divided into three 10-minute periods, with the lights turned on only during the second period. This sequence was repeated on 4 consecutive days. In one study, the local anesthetics lidocaine (2%) and the charged lidocaine derivative N-ethyl bromide (QX-314, 0.5%) were co-applied to the cornea (15 µl) 3 minutes before the start of each session to examine the role of corneal afferent activity in conditioned learning. The ability of lidocaine to transiently open TRPV1 channels was utilized to permit the entry of QX-314 into nociceptor terminals, resulting in sustained anesthesia.^32^

### Palpebral Opening

Eye closure was measured using a ratio consisting of the height of the gap between the upper and lower eyelids and the distance separating the two canthi, as previously described.^15^ The effect of ArchT activating light on palpebral opening was examined by placing mice in an enclosed Plexiglas chamber (12 cm × 12 cm × 20 cm) with transparent sides for 20 minutes. After a 5-minute acclimation period, baseline palpebral opening was measured from 5 still images taken from video over a 5-minute period. Mice were then exposed to ArchT activating light (35–45 lux) for 10 minutes, with 5 still images taken during the final 5 minutes. Using the same chamber, the palpebral opening was also used to determine the duration of corneal anesthesia after co-application of QX-314 and lidocaine.

### Corneal Fluorescein

In a subgroup of C57BL/6J mice, corneal fluorescein staining was performed after exposure to either the ArchT activating light or the control light to examine the effect on the integrity of the corneal epithelium. Animals were placed in an enclosed Plexiglas chamber (12 cm × 12 cm × 20 cm) with transparent sides and exposed to the LED light source for 30 minutes. Under isoflurane anesthesia, a 1% fluorescein solution was applied to the cornea and the cornea was examined with cobalt blue light, as previously described.^15^^,^^31^

### Statistical Analysis

Data were analyzed to confirm normal distribution and equal variance prior to conducting statistical comparisons. Multiple group comparisons were performed using 1-way or 2-way ANOVAs with repeated measures when appropriate. Comparisons between only two treatment groups were made using the paired sample *t*-test. The Holm-Sidak's post hoc multiple comparison test was performed to examine individual comparisons between the groups and for comparisons with baseline. Analyses were performed using commercial software (GraphPad Prism 9; GraphPad Software, San Diego, CA, USA). All results are expressed as mean ± SEM. Values of *P* < 0.05 were considered to be statistically significant.

## Results

### ArchT/eGFP Expression in Corneal Afferents

**Figure 1.**
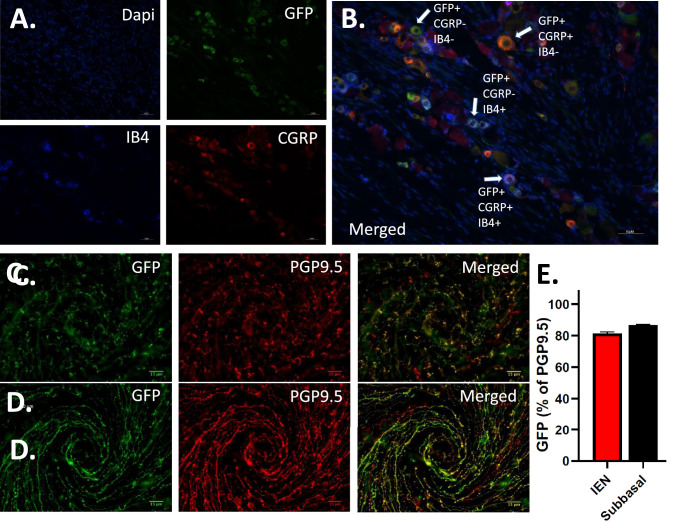
ArchT/eGFP expression in the trigeminal ganglion and corneal nerve fibers in Nav1.8-cre;ArchT/eGFP mice. **(A**) Representative images from a trigeminal ganglion section with eGFP positive cells (*upper right*) after labeling for cell nuclei (DAPI, *top left*), IB4 (*lower left*), and CGRP (*lower right*). (**B**) Merging the images from **A** shows significant overlap of eGFP with both IB4 and CGRP. (**C**) A whole mounted cornea showing eGFP and PGP9.5 labeling of the intraepithelial nerve (IEN) endings. (**D**) The eGFP and PGP9.5 subbasal nerve innervation in the cornea. (**E**) Quantification of eGFP in the intraepithelial and subbasal corneal nerves as a percentage of total innervation using the pan neuronal marker PGP9.5 (*n* = 6 corneas, 3 male and 3 female mice).

Neuronal cell bodies in the trigeminal ganglion from Nav1.8-cre;ArchT/eGFP mice showed extensive labeling with eGFP (Fig. 1). Sections were labeled for CGRP and IB4 to confirm the presence of eGFP in peptidergic and non-peptidergic C-fibers. Indeed, most neurons were labeled with IB4, CGRP, or infrequently both IB4 and CGRP (see Figs. 1A, 1B), indicating expression of Archt/eGFP in non-peptidergic and peptidergic nociceptors. Previously, we have shown that Nav1.8-cre;tdTomato reporter mice demonstrated robust labeling of corneal afferent nerve fibers in both the subbasal nerve plexus and intraepithelial nerve endings.^15^ Whole corneas were examined to confirm successful expression of eGFP in Nav1.8-cre;ArchT/eGFP mice in neurons innervating the cornea. Robust eGFP signal was found in both the intraepithelial nerve endings (see Fig. 1C) and subbasal nerves (see Fig. 1D). Immunohistochemistry with an antibody for the pan-neuronal marker PGP9.5 was used to determine the relative overlap of eGFP with all corneal nerve innervation (*n* = 6 corneas, 3 male and 3 female mice). An average of 81 ± 1.2% of PGP9.5 labeled axons overlapped with eGFP within the intraepithelial nerve endings, and 86 ± 0.6% overlapped in the subbasal nerve bundles (see Figs. 1C–E).

### ArchT Activating Light Produces Real Time Place Preference Following LGE

**Figure 2.**
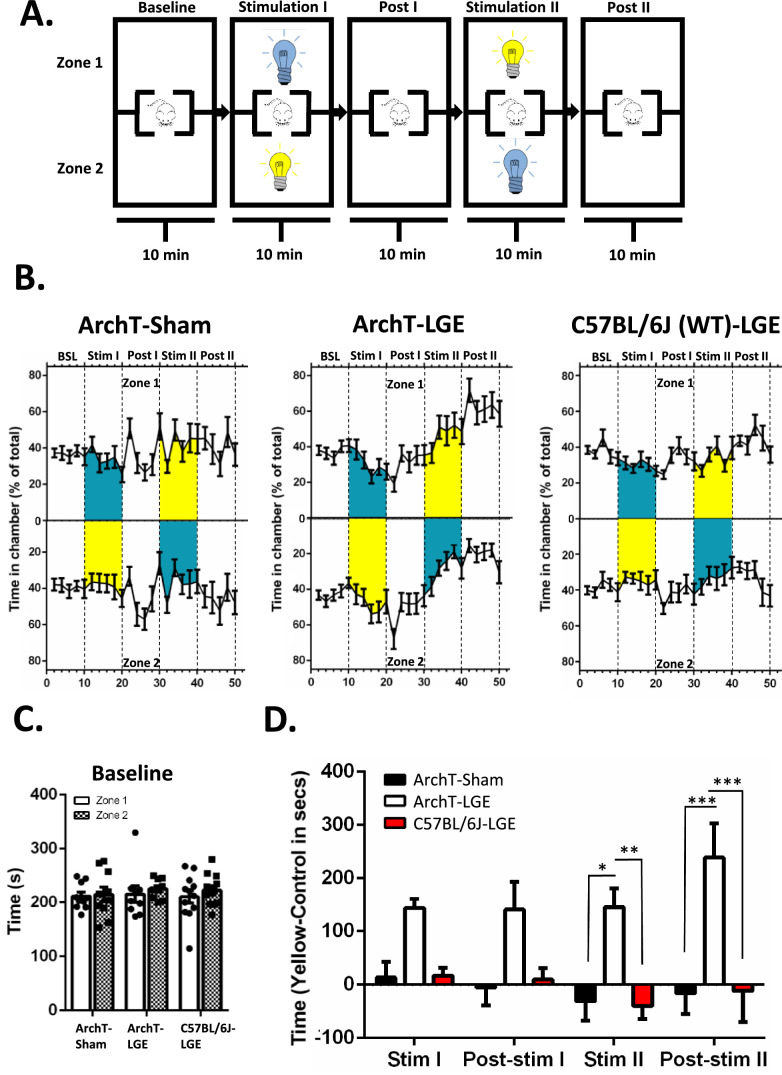
Real-time place preference in tear deficient mice. (**A**) Illustration of the active learning paradigm showing the box and light setup for the 50-minute session. The compartments illuminated with the yellow and control lights were switched between stimulation I and stimulation II. (**B**) The percent time mice spent in each chamber during each of the five 10-minute periods across the 50-minute session. ArchT expressing mice tested after lacrimal gland excision (LGE) spent more time in the ArchT-activating light chamber, especially evident during the latter part of the second stimulation period and during the final post-stimulation period. ArchT expressing mice with sham surgery and C57BL/6J wild type mice with LGE did not develop this preference. (**C**) Mice spent similar amounts of time in each of the two zones during the baseline period (first 10-minute session). (**D**) ArchT expressing mice with LGE showed a robust preference for the chamber illuminated with ArchT-activating light by the second stimulation period which remained even after the light was turned off (post-stimulation II). The Y-axis is average time (seconds) spent in the yellow paired chamber minus the time spent in control light chamber. BSL, baseline. * *P* < 0.05; ** *P* < 0.01; *** *P* < 0.001; *n* = 10 Nav1.8;ArchT sham (5 female and 5 male mice), 9 Nav1.8;ArchT LGE (5 female and 4 male mice), 12 C57BL/6J (6 female and 6 male mice).

To test for real time learning, a single 50-minute session was performed, consisting of five 10-minute periods: baseline, stimulation 1, post-stimulation 1, stimulation 2, and post-stimulation 2 (Fig. 2A). As illustrated in Figure 2A, the ArchT activating LED was turned on in periods 2 and 4, but in the alternate compartments. Over the 50-minute session, ArchT mice with sham surgery did not develop a preference for the ArchT activating LED paired compartment (Fig. 2B). However, ArchT mice that received LGE 2 weeks prior to testing developed a clear preference for the ArchT activating light. A gradual shift was observed in time spent in the ArchT activating LED chamber across the 10-minute period of light exposure, which is especially prominent during the second period (stimulation 2, see Fig. 2B). Of note, animals consistently showed the greatest preference for the ArchT activating LED paired chamber immediately after the ArchT activating light was turned off, especially during the first 2 minutes of the post-stimulation periods. No preference for either zone developed in LGE treated C57BL/6J mice.

Analysis of the average time mice spent in the two chambers during the baseline acclimation period indicated no inherent preference for either zone, with all three treatment groups (ArchT-sham, ArchT-LGE, and C57BL/6J-LGE) exploring each chamber for similar amounts of time before exposure to the lights (Fig. 2C, 2-way ANOVA, *P* > 0.05; Table 1). The difference in time spent in the yellow-illuminated chamber and the control chamber for each time period, beginning with the first light stimulation period, was calculated to compare the effect of ArchT activating light on behavior between the different treatment groups (Fig. 2D). During each time period, only the ArchT-LGE animals demonstrated a clear preference for the chamber paired with the ArchT activating light (see Fig. 2D, 2-way ANOVA with repeated measures, see Table 1). Post hoc analysis indicated that ArchT mice with LGE spent more time in the ArchT activating chamber during and after the second stimulation period when compared to the ArchT sham and C57BL/6J-LGE control groups (see Fig. 2D).

**Table 1.** Results From 2-Way ANOVA Statistical Analysis for All Data Sets

		Zone (Zone 1/Zone 2)			Surgery/Genotype			Interaction		
Figure	Measurement (Units)	DFn, DFd	F	*P* Value	DFn, DFd	F	*P* Value	DFn, DFd	F	*P* Value
2C	Time (s)	1, 56	0.8592	<0.3579	2, 56	0.2002	<0.8192	2, 56	0.08595	<0.9178

		**Period**			**Surgery/genotype**			**Interaction**		
2D	Time (s)	3, 112	0.7857	0.5043	2, 112	28.88	<0.0001	6, 112	0.6997	0.6504

		**Treatment**			**Time**			**Interaction**		
3B	Palpebral opening	2, 22	46.51	<0.0001	7, 154	14.73	<0.0001	14, 154	11.94	<0.0001

The two independent factors included zone (zone 1 and zone 2) and surgery/genotype (ArchT/sham, ArchT/LGE, and C57BL-6J/LGE), period (stimulation I, post-stimulation I, stimulation II, and post-stimulation II) and surgery/genotype, or treatment (QX-314+lidocaine and QX-314, lidocaine) and time (minutes).

DFn, degrees of freedom numerator; DFd, degrees of freedom denominator; F, F statistic.

To determine whether the integrity of the corneal epithelium was affected by exposure to ArchT-activating light or the control light, fluorescein was applied to the cornea after 30 minutes of light exposure in C57BL/6J mice (*n* = 6). There was an absence of corneal fluorescein staining in all animals, indicating that the ArchT-activating light and control light had no adverse effect on the corneal epithelium.

### The Effect of ArchT Activating Light on the Palpebral Opening

**Figure 3.**
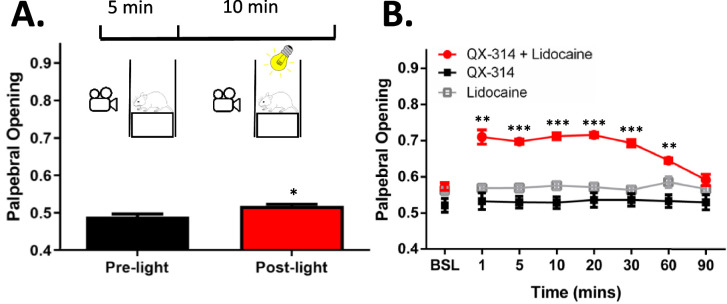
Palpebral opening after exposure to ArchT-activating light and corneal application of a local anesthetic. (**A**) Light stimulation produced a small but significant increase in palpebral opening in tear deficient ArchT expressing mice. *N* = 8 (4 female and 4 male mice). (**B**) Co-application of local anesthetic QX-314 and lidocaine increased palpebral opening for an extended period of time when compared to QX-314 or lidocaine alone. *N* = 8 QX-314 + lidocaine (4 female and 4 male mice), 6 QX-314 (3 female and 3 male mice), and 11 lidocaine (6 female and 5 male mice). * *P* < 0.05; ** *P* < 0.01; *** *P* < 0.001.

Lacrimal gland excision causes squinting in the affected eye, which we previously have shown can be reversed by corneal application of local anesthetics.^15^ ArchT activating light was used to determine the effect of selectively inhibiting Nav1.8-expressing neurons on palpebral opening after LGE. Only a nominal albeit significant increase in palpebral opening was observed after 5 minutes of exposure to ArchT activating light (Fig. 3A, paired sample *t*-test, *P* < 0.05). As a comparison, palpebral opening was quantified after the co-application of the charged lidocaine derivative QX-314 and lidocaine. In contrast to the minor effect of ArchT activating light on palpebral opening, co-application of QX-314 and lidocaine produced a robust and long-lasting reversal of the decrease in palpebral opening caused by LGE (Fig. 3B, 2-way ANOVA with repeated measures, see Table 1). In contrast, neither lidocaine nor QX-314 applied alone produced any change in palpebral opening when compared to baseline values (see Fig. 3B).

### ArchT Activating Light Produces Conditioned Place Preference Over Multiple Sessions

**Figure 4.**
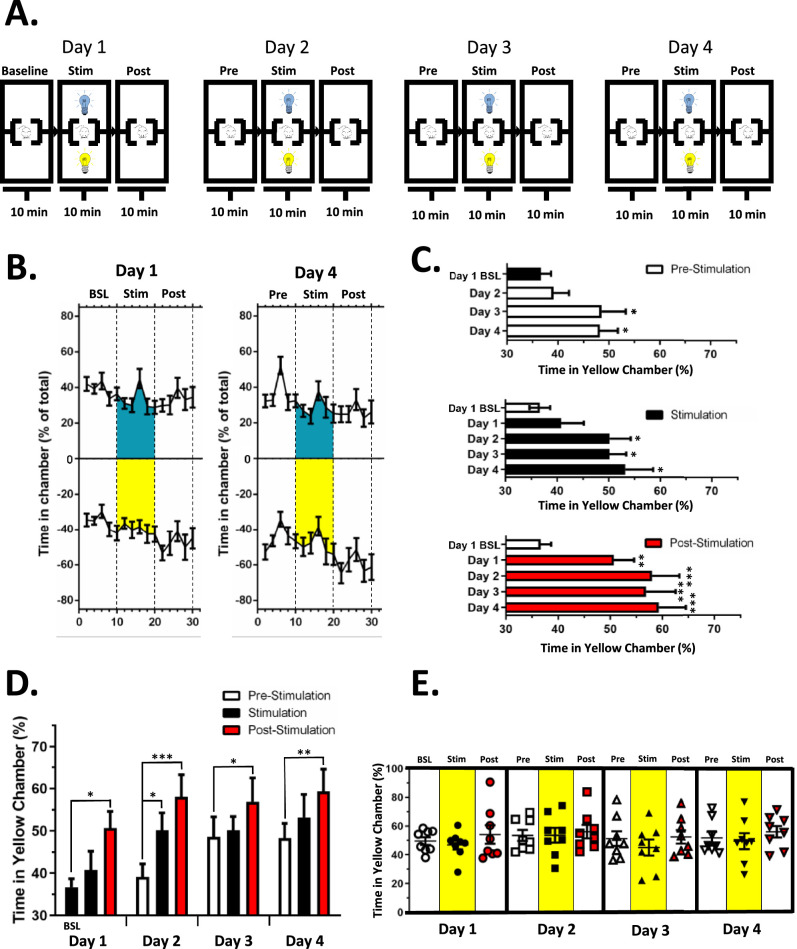
Conditioned place preference in tear deficient mice. (**A**) The 4-day conditioning paradigm illustrating each 30-minute session. (**B**) The average time ArchT mice with LGE spent in ArchT-activating light and control chambers during the first and fourth sessions. Of note, at the start of the fourth session, mice spent more time in the chamber where light stimulation was given on the previous days. (**C**) A comparison of the day 1 baseline (BSL) time in the “yellow” compartment with time spent in this compartment during the prestimulation period (*top panel*), stimulation period (*middle panel*), and post-stimulation period (*bottom panel*) across the 4-day experiment. By the third day, mice spent more time in the ArchT-activating light paired compartment even during the prestimulation period. (**D**) Across the 4-day experimental paradigm, mice developed a preference for the ArchT-activating light-paired chamber throughout the entire session, which can be seen as loss of significance between the pre-stimulation period and the stimulation and post-stimulation periods beginning on day 3. * *P* < 0.05; ** *P* < 0.01; *** *P* < 0.001; *n* = 10 (5 male and 5 female mice). (**E**) Tear deficient ArchT mice pretreated with co-application of QX-314 and lidocaine prior to each session failed to develop a conditioned preference for the ArchT-activating light paired chamber; *n* = 8 (4 male and 4 female mice).

Conditioned learning was examined across 4 separate sessions run on consecutive days 2 weeks after LGE in Nav1.8-cre;ArchT mice (Fig. 4A). On each day, animals were placed in a 3-chambered apparatus and activity tracked across three 10-minute testing periods. The LEDs were off during the initial 10 min, which was followed by a second 10-minute period when 2 of the chambers were illuminated with either an ArchT activating or control LED. This was followed by a final 10-minute period in which the LED light sources were turned off (see Fig. 4A). Of note, the chamber illuminated with the ArchT activating LED remained the same across all 4 days. Tracking of the mice showed a gradual increase in the percentage of time spent in the ArchT activating LED illuminated chamber across the 10-minute stimulation period, with an even greater increase observed on the fourth day of testing when compared to the first day (Fig. 4B). As with the single-day real-time place preference paradigm, the initial 2-minute period post-LED stimulation showed the greatest preference for the ArchT activating LED paired chamber (see Fig. 4B).

A comparison of the overall percentage of time spent in the ArchT activating chamber during the initial baseline period on day 1, just prior to the light exposure, with the first 10 minutes on days 2 to 4 indicated a shift across the 4 days with animals spending significantly more time in the ArchT activating LED chamber on days 3 and 4 (Fig. 4C, *P* < 0.05 compared to day 1 baseline, 1-way ANOVA, Table 2). Likewise, a similar trend was found during the stimulation period, with an increase in the percentage of time spent in ArchT activating LED chamber first developing on the second day of testing (see Fig. 4C, *P* < 0.05 compared to day 1 baseline, 1-way ANOVA, see Table 2). The most robust effect was observed during the final period of the session after the ArchT activating LED was turned off. At this time, a preference for the ArchT activating chamber developed on the first day (see Fig. 4C, *P* < 0.01 compared to day 1 baseline, 1-way ANOVA, see Table 2).

**Table 2.** Results From 1-Way ANOVA Statistical Analysis for All Data Sets

Figure	Time Period	DFn, DFd	F	*P* Value
4C	Prestimulation	3, 27	3.798	0.0217
4C	Stimulation	4, 36	3.855	0.0104
4C	Post-stimulation	4, 36	6.615	0.0004
4D	Day 1	2, 18	5.044	0.0182
4D	Day 2	2, 18	10.73	0.0009
4D	Day 3	2, 18	5.454	0.0141
4D	Day 4	2, 18	6.800	0.0063
4E	Day 1	2, 14	0.3641	0.7012
4E	Day 2	2, 14	0.07774	0.9256
4E	Day 3	2, 14	1.416	0.2753
4E	Day 4	2, 14	0.7271	0.5007

A comparison of time spent in the ArchT activating light chamber during the 3 time periods on each training day illustrates the learning that occurred across the 4 days (Fig. 4D). The mice spent a significantly greater percentage of time in the yellow light-illuminated chamber by the second day of exposure compared to the prestimulation period on the same day (see Fig. 4D, *P* < 0.05, 1-way ANOVA, see Table 2). However, no difference was observed on subsequent days, due to the elevation in the percentage of time the mice spent in the ArchT activating light chamber during the prestimulation period on the third and fourth days of exposure (see Fig. 4D). Also evident is the greater level of preference observed during the post-stimulation period, which remained elevated compared to the prestimulation period even on day 4 (see Fig. 4D).

### Local Anesthesia Prevents Conditioned Place Preference in Tear Deficient Nav1.8-cre;ArchT Mice

If inhibition of corneal nociceptors by ArchT activating light is required for establishing place preference, then pre-emptive inhibition of nociceptors by corneal application of a local anesthetic should prevent ArchT-mediated place preference. The co-application of QX-314 and lidocaine was utilized to provide full anesthesia for the duration of each 30-minute session across each of the 4 days. Topical corneal application of QX-314 and lidocaine was administered 3 minutes prior to the start of each conditioning session in Nav1.8-cre;ArchT mice 2 weeks after LGE. Following QX-314 and lidocaine pretreatment, the time spent in the ArchT light activating chambers remained constant over the four sessions, indicating that the QX-314 and lidocaine completely prevented place preference learning (Fig. 4E, 1-way ANOVA, see Table 2).

## Discussion

The presence of ongoing pain in an LGE model of persistent aqueous tear deficiency was examined using a place preference paradigm in Nav1.8-cre;ArchT/eGFP transgenic mice. A real-time conditioned place preference was produced with learning occurring during a single session that consisted of ArchT activating light exposure during two 10-minute periods. Furthermore, a robust conditioned place preference was achieved with single sessions across 4 days, in which tear deficient mice learned to prefer the light paired chamber by the end of the second day. Furthermore, application of a local anesthetic to the cornea prevented conditioned learning, suggesting that ongoing corneal nociceptor activity was necessary for the light-induced preference to occur.

Two different control groups were included to ensure that the behavioral effect of ArchT activating light was specific to LGE treated animals expressing ArchT. C57/B6 wild-type control mice did not demonstrate a preference for the ArchT-activating light paired chamber following LGE, indicating the necessity of ArchT expression in Nav1.8-positive neurons. In addition, Nav1.8-cre;ArchT/eGFP sham treated animals showed no preference for the ArchT-activating light paired chamber, indicating the necessity of corneal pain. Likewise, aversion to the control LED was not found in either of these experimental control groups.

The cornea is the mostly densely innervated tissue in mammals consisting exclusively of C-fibers and A-delta fibers (approximately 70% c-fibers and 30% A-delta fibers, in a mouse), which respond to chemical, thermal, mechanical, and environmental stimuli.^33^ The voltage-gated sodium channel Nav1.8 is preferentially expressed in small-diameter unmyelinated sensory afferents in the dorsal root ganglion (DRG), with >90% of IB4-binding neurons (nonpeptidergic C-nociceptors) and CGRP-positive and substance P-positive neurons (peptidergic C-fibers) expressing tdTomato fluorescent protein in Nav1.8-cre;tdTomato Cre-reporter mice.^28^ Of note, a significant portion of A-beta and A-delta DRG neurons, approaching 40%, also were positive for Nav1.8-cre.^28^ Consistent with these findings, characterization of trigeminal ganglion neurons in Nav1.8-cre;ArchT/eGFP mice showed robust labeling of cell bodies colocalized with IB4 and CGRP. Furthermore, we found that greater than 80% of both the corneal subbasal nerve and intraepithelial nerve endings were robustly labeled with eGFP. These results, taken together with a previous study reporting over half of mouse corneal afferents are peptidergic (CGRP or substance P positive), indicate that a large portion of the neurons expressing ArchT are peptidergic nociceptors.^34^ The localization of eGFP in the corneal nerve terminals would also allow for an illuminated chamber to activate ArchT.

Previous studies have used optogenetics to activate select populations of primary afferent neurons, leading to insights into their function.^26^^,^^35^^–^^40^ Using the Nav1.8-cre mouse to drive ChR2 expression, optical stimulation of the hindpaw in mice elicited paw withdrawal, licking, jumping, and vocalizations. In addition, a conditioned place aversion was produced using a pulsed light through a glass floor. These responses could all be mitigated by pretreatment with morphine.^26^ Optogenetic activation of TRPV1 positive peptidergic C-fibers innervating the hindpaw also produced a vigorous withdrawal response and conditioned place aversion, whereas optical activation of MrgD positive, nonpeptidergic cutaneous afferents evoked a less robust hindpaw withdrawal reaction and failed to induce a conditioned place aversion.^36^ A similar optogenetic approach was used to elicit nociceptive responses by activating a subpopulation of A-delta fibers and demonstrate a role of low threshold A-beta fibers in nerve injury-induced pain.^35^^,^^40^

Other studies have utilized archearodhopsin-3 and ArchT to inhibit subpopulations of primary afferent neurons.^26^^,^^27^^,^^41^^,^^42^ Optical inhibition of Nav1.8 positive cutaneous c-fibers reduced mechanical hypersensitivity in inflammatory and neuropathic pain models but had only minimal effects on sensitivity to heat.^26^ Similar to the effects of inhibiting Nav1.8 positive neurons, optical inhibition of CGRP-alpha neurons reduced mechanical hypersensitivity following inflammation and nerve injury.^29^ Furthermore, inhibition of CGRP-alpha expressing primary afferents increased heat withdrawal latencies whereas cold latencies decreased. Optical inhibition has also been used to demonstrate a contribution of myelinated high threshold mechanoreceptors to nerve injury-induced mechanical hypersensitivity.^41^

In addition to examining nociceptive reflexes, optical inhibition of peripheral terminals of nociceptors has been used to produce real time conditioned place preference after injury. In a mouse model of bladder pain, a real time place preference was produced by optical inhibition of Nav1.8 positive afferent terminals over a single 20-minute session, providing evidence for ongoing bladder pain.^42^ We found a similarly robust place preference produced in Nav1.8-cre;ArchT mice after LGE, indicating that persistent peripheral nociceptor activation drives ongoing pain in this model of dry eye. Peripheral inhibition of cutaneous CGRP-alpha neurons also produced a real time place preference following spinal nerve ligation.^27^ In this study, a single 30-minute session was performed consisting of six 5-minute periods alternating between light off and light on, with significant preference observed by the third and final 5-minute light on period. Although place preference developed during the second light period in our study, the longer periods of light exposure (10 minutes versus 5 minutes) likely account for this difference.

Although LGE produces severe dry eye typically not observed in the clinic, it serves as an additional utility as a model for chronic, ongoing corneal pain and can be useful in identifying novel targets to treat this pain. Previous studies have quantified palpebral opening to assess the ongoing pain after LGE.^9^^,^^15^ The contribution of persistent nociceptor activity in driving LGE-induced decreases in palpebral opening was demonstrated using local anesthetics applied to the cornea. Compared to the local anesthetic QX-314 plus lidocaine, ArchT activating light produced only minimal effects on palpebral opening. It is possible that the optical inhibition did not sufficiently suppress neuronal activity to fully rescue the palpebral opening, a possibility made more likely by the severity of corneal injury produced by LGE. However, these results also suggest that the aversive quality of ocular pain is more sensitive to the inhibition of corneal nociceptors than motor reflex behaviors (i.e. reduction in palpebral opening). Alternatively, the change in palpebral opening may be driven by a distinct set of corneal afferents that do not express Nav1.8.

A multi-day conditioned learning paradigm was developed in order to allow for a complete local anesthetic block that lasted for the duration of the testing session. Various local anesthetics have been utilized to rescue palpebral opening, yet all had a relatively short duration of action (<30 minutes).^15^ Corneal anesthesia with QX-314 plus lidocaine began to dimmish 1 hour after application, far longer than other local anesthetics tested in the LGE model. QX-314, a charged voltage gated sodium-channel blocker, can be used to target nociceptors when applied with a TRPV1 channel agonist, thereby allowing for entry of QX-314 into the axon terminal and providing prolonged anesthesia.^43^ Lidocaine, which in addition to blocking voltage gated sodium channels activate TRPV1,^44^ has been used previously with QX-314 to allow for entry of QX-314 into nociceptor terminals.^32^

The ability of a local anesthetic to prevent conditioned learning over the course of the 4 days indicates that ongoing corneal nociceptor activity is necessary for light-induced preference to occur. A similar strategy has been used to demonstrate the contribution of peripheral nociceptive input to persistent, ongoing pain produced by knee osteoarthritis, bone cancer, and inflammation.^45^^–^^48^ This approach has also been used to assess spinal analgesics and brain regions, such as the rostral ventromedial medulla, that may contribute to the aversive state of ongoing pain.^25^^,^^49^ Conditioned place preference produced after LGE-induced dry eye provides the opportunity to explore the contribution of specific corneal nociceptor subtypes and central neural circuits to the tonic aversive state caused by chronic ocular pain.
